# Clinicopathological Analysis of Lipomatous Tumors in a Tertiary Care Center

**DOI:** 10.7759/cureus.110579

**Published:** 2026-06-10

**Authors:** Divya Maheshwari, Lawrence D'Cruze, Banukeerthana R, Subalakshmi Balasubramanian

**Affiliations:** 1 Pathology, Sri Ramachandra Institute of Higher Education and Research, Chennai, IND

**Keywords:** atypical lipomatous tumor, dedifferentiated liposarcoma, histopathology, lipoma, liposarcoma, mdm2/cdk4, soft tissue tumors, subcutaneous tumors, tumor size

## Abstract

Background and aim

Adipocytic tumors are among the most common soft tissue neoplasms, ranging from benign to malignant forms. Accurate differentiation between these entities is essential due to differences in prognosis and management. This study aims to evaluate the clinicopathological spectrum of lipomatous tumors, including their demographic profile, anatomical distribution, and histopathological spectrum, and to analyze factors associated with malignancy.

Methods

A retrospective observational study was conducted in the Department of Pathology at a tertiary care center from October 2019 to September 2025. A total of 114 histopathologically confirmed cases of adipocytic tumors were included. Clinical and pathological data were retrieved and analyzed. Tumors were classified according to the WHO classification. Ancillary techniques, including immunohistochemistry (MDM2 and CDK4), were performed in selected cases. Associations between tumor size, site, radiological features, and malignancy were assessed.

Results

The majority of patients were in the fourth to sixth decade age group (52 cases, 45%), with a male predominance (68 cases, 60%). The upper extremity was the most common site (32 cases, 28%), followed by the lower extremity (30 cases, 26%) and trunk (28 cases, 25%). Conventional lipoma was the most common tumor type (72 cases, 63%), followed by other variants such as fibrolipoma (12 cases, 11%) and angiolipoma (10 cases, 9%). The most common anatomical location was subcutaneous tissue, followed by deep soft tissue and intramuscular locations. Atypical lipomatous tumor/well-differentiated liposarcoma and myxoid liposarcoma were identified in a subset of cases. Radiologically, benign lesions showed homogeneous fat density, whereas malignant tumors demonstrated septal thickening, nodularity, and non-adipose components. Most tumors measured less than 5 cm (64 cases, 56%). MDM2/CDK4 immunohistochemistry findings indicate that atypical/malignant adipocytic neoplasms were positive in four cases.

Conclusions

Lipomatous tumors are predominantly benign, with lipoma being the most common subtype. Tumor size and location serve as important indicators of malignancy. Comprehensive histopathological evaluation, supplemented by ancillary techniques where necessary, is crucial for accurate diagnosis and appropriate management.

## Introduction

Lipomatous tumors represent one of the most common categories of mesenchymal neoplasms, encompassing a wide spectrum ranging from benign lipomas to malignant liposarcomas. According to the WHO classification of soft tissue tumors, these neoplasms are categorized based on their morphological, immunohistochemical, and molecular characteristics, reflecting their diverse biological behavior and clinical significance [1]. While benign lipomas constitute the majority of cases encountered in routine clinical practice, distinguishing them from atypical and malignant counterparts remains a critical diagnostic challenge.

Recent advances have led to the recognition of new entities and evolving concepts in the classification of liposarcomas, particularly atypical lipomatous tumors (ALTs) and well-differentiated liposarcomas (WDLs), which share overlapping histological features with benign lipomas but differ significantly in their clinical course and management [2]. The differentiation between these entities is essential, as ALT/WDLS may demonstrate local recurrence and, in certain cases, progression to dedifferentiated liposarcoma.

Ancillary diagnostic techniques have significantly improved the accuracy of classification. Immunohistochemical markers such as MDM2 and CDK4 have been widely used to distinguish benign lipomas from ALT/WDLS. Furthermore, molecular techniques, particularly fluorescence in situ hybridization (FISH) for MDM2 gene amplification, have emerged as a gold standard for confirming the diagnosis of atypical and malignant lipomatous tumors [3-5]. These advances highlight the importance of integrating histopathological findings with molecular diagnostics in contemporary practice.

Clinically, lipomatous tumors present across a broad age range, with a predilection for middle-aged and older adults. They most commonly arise in the extremities and trunk, although deep-seated lesions, including retroperitoneal tumors, are of particular concern due to their association with malignancy [6,7]. Imaging modalities such as MRI play a crucial role in preoperative evaluation, aiding in the differentiation between benign and malignant lesions based on characteristic radiological features [8-10].

Tumor size and anatomical location have been identified as important predictors of malignancy. Larger tumors, especially those located in deep compartments, are more likely to represent ALT or liposarcoma, necessitating careful evaluation and management [6,10]. Additionally, subtype-specific prognostic models have been developed to better understand the clinical behavior and outcomes associated with different categories of liposarcoma [10,11].

From a pathological standpoint, the spectrum of lipomatous tumors continues to evolve with updates in classification systems. The WHO 2020 classification emphasizes the integration of morphological features with molecular alterations to achieve precise diagnosis [12-14]. Ongoing research has also focused on developing diagnostic models combining clinical, radiological, and molecular parameters to improve differentiation between benign and malignant lipomatous tumors [14,15].

Despite these advances, challenges remain in accurately diagnosing and categorizing lipomatous tumors, particularly in resource-limited settings where access to molecular testing may be restricted. Therefore, clinicopathological studies evaluating the distribution, histological spectrum, and malignancy findings are essential to enhance diagnostic accuracy and guide appropriate management. The present study aims to analyze the clinicopathological profile of lipomatous tumors in a tertiary care center, with particular emphasis on their histopathological spectrum, anatomical distribution, and the association between tumor size and malignancy.

## Materials and methods

This retrospective observational study was conducted in the Department of Pathology at Sri Ramachandra Medical College and Research Institute, Chennai. The study period extended from October 2019 to September 2025. The study population comprised all cases of lipomatous tumors that were histopathologically diagnosed during the specified period.

Cases were included if they met the following criteria: histologically confirmed diagnosis of lipomatous tumors, availability of adequate tissue for evaluation, and complete clinical data. Cases with inadequate tissue samples or those diagnosed as non-lipomatous soft tissue tumors were excluded from the study.

Data were retrieved from the laboratory information system and relevant medical records. The variables collected included patient demographics such as age and gender, tumor characteristics including size, anatomical site, and multiplicity, as well as radiological findings wherever available. Histopathological subtypes were also documented for all cases.

All tissue specimens were processed using standard protocols. Formalin-fixed, paraffin-embedded sections were stained with hematoxylin and eosin and reviewed independently. Tumors were classified according to the WHO classification criteria for soft tissue tumors [1].

Statistical analysis was performed using descriptive methods, with results expressed as frequencies and percentages. Ethical approval considerations were addressed appropriately. The study utilized archived, anonymized samples, ensuring patient confidentiality. As this was a retrospective study using existing data, a waiver of informed consent was obtained.

## Results

A total of 114 cases of histopathologically confirmed lipomatous tumors were included in the study. The demographic profile of the study population is summarized in Table 1. The majority of patients belonged to the 41-60 years age group (45%), followed by the 21-40 years age group (33%). Patients aged over 60 years constituted 15% of cases, while those younger than 20 years accounted for 7%. There was a male predominance, with 68 cases (60%), whereas females comprised 46 cases (40%).

**Table 1:** Demographic characteristics of study population

Variable	Number (n)	Percentage (%)
Age (years)		
<20	8	7%
21-40	38	33%
41-60	52	45%
>60	16	15%
Gender		
Male	68	60%
Female	46	40%
Total	114	100%

The anatomical distribution of tumors is presented in Table 2. The upper extremity was the most common site of involvement, accounting for 28% of cases, followed by the lower extremity (26%), trunk (25%), and head and neck region (12%). Deep-seated tumors, including retroperitoneal locations, constituted 9% of cases.

**Table 2:** Anatomical distribution of lipomatous tumors

Site	Number (n)	Percentage (%)
Upper extremity	32	28%
Lower extremity	30	26%
Trunk	28	25%
Head and neck	14	12%
Retroperitoneum/deep sites	10	9%
Total	114	100%

The histopathological spectrum of lipomatous tumors is detailed in Table 3. Conventional lipoma was the most frequently encountered subtype, comprising 63% of cases. Among the variants, fibrolipoma (11%) and angiolipoma (9%) were the most common. Myolipoma accounted for 4% of cases. Malignant or borderline tumors included ALT in 5% and WDLs in 4% of cases. Tumor size distribution is shown in Table 4. The majority of tumors measured less than 5 cm (56%), followed by those measuring 5-10 cm (26%). Larger tumors exceeding 10 cm constituted 18% of cases.

**Table 3:** Histopathological spectrum of lipomatous tumors Percentages may not total 100% due to rounding. ALT, atypical lipomatous tumor; WDL, well-differentiated liposarcoma

Tumor type	Number (n)	Percentage (%)
Lipoma	72	63%
Fibrolipoma	12	11%
Angiolipoma	10	9%
Myolipoma	4	4%
ALT	6	5%
WDL	4	4%
Myxoid liposarcoma	3	3%
Dedifferentiated liposarcoma	3	3%
Total	114	100%

**Table 4:** Tumor size distribution

Tumor size	Number (n)	Percentage (%)
<5 cm	64	56%
5-10 cm	30	26%
>10 cm	20	18%
Total	114	100%

The relationship between tumor size and malignancy is summarized in Table 5. Most benign tumors were less than 5 cm in size (62 out of 64 cases), whereas malignant tumors (ALT/WDLS) were more frequently associated with larger sizes. Notably, among tumors larger than 10 cm, malignant cases (n = 10) were more common compared to smaller size categories. This suggests that increasing tumor size is associated with a higher likelihood of malignancy.

**Table 5:** Distribution of benign and malignant lipomatous tumors according to tumor size ^*^ Malignant lipomatous tumors included ALT, WDL, dedifferentiated liposarcoma, and myxoid liposarcoma. ALT, atypical lipomatous tumor; WDL, well-differentiated liposarcoma

Tumor size	Benign (n)	Malignant lipomatous tumors^*^ (n)	Total
<5 cm	62	2	64
5-10 cm	24	6	30
>10 cm	10	10	20
Total	96	18	114

Radiological evaluation showed concordance with histopathological diagnosis in lipomatous tumors. Benign lesions such as lipomas typically exhibit homogeneous fat density, thin septations, and absence of contrast enhancement, reflecting their composition of mature adipose tissue. In contrast, atypical and malignant tumors showed characteristic features including thickened septa (>2 mm), nodular non-adipose components, heterogeneous signal intensity, and contrast enhancement, often located in deeper anatomical planes. These radiological features showed an increased frequency of malignancy, particularly in tumors larger than 10 cm and those arising in deep-seated locations. Furthermore, imaging heterogeneity and the presence of non-fatty areas are associated with higher-grade histology.

Higher-grade lesions such as dedifferentiated liposarcoma show heterogeneous signal intensity with prominent non-adipose solid components and significant enhancement, indicating areas of high cellularity and pleomorphism. Similarly, myxoid liposarcomas demonstrate a characteristic intermediate to low T1 signal and high T2 signal intensity with a myxoid matrix, often with a plexiform vascular pattern, correlating with abundant myxoid stroma on histology. Additionally, radiological features such as deep anatomical location, large tumor size (>10 cm), and infiltrative margins are strongly associated with malignancy. Thus, radiological assessment not only aids in lesion detection and surgical planning but also serves as a critical adjunct to histopathology in differentiating benign from malignant lipomatous tumors.

**Figure 1:**
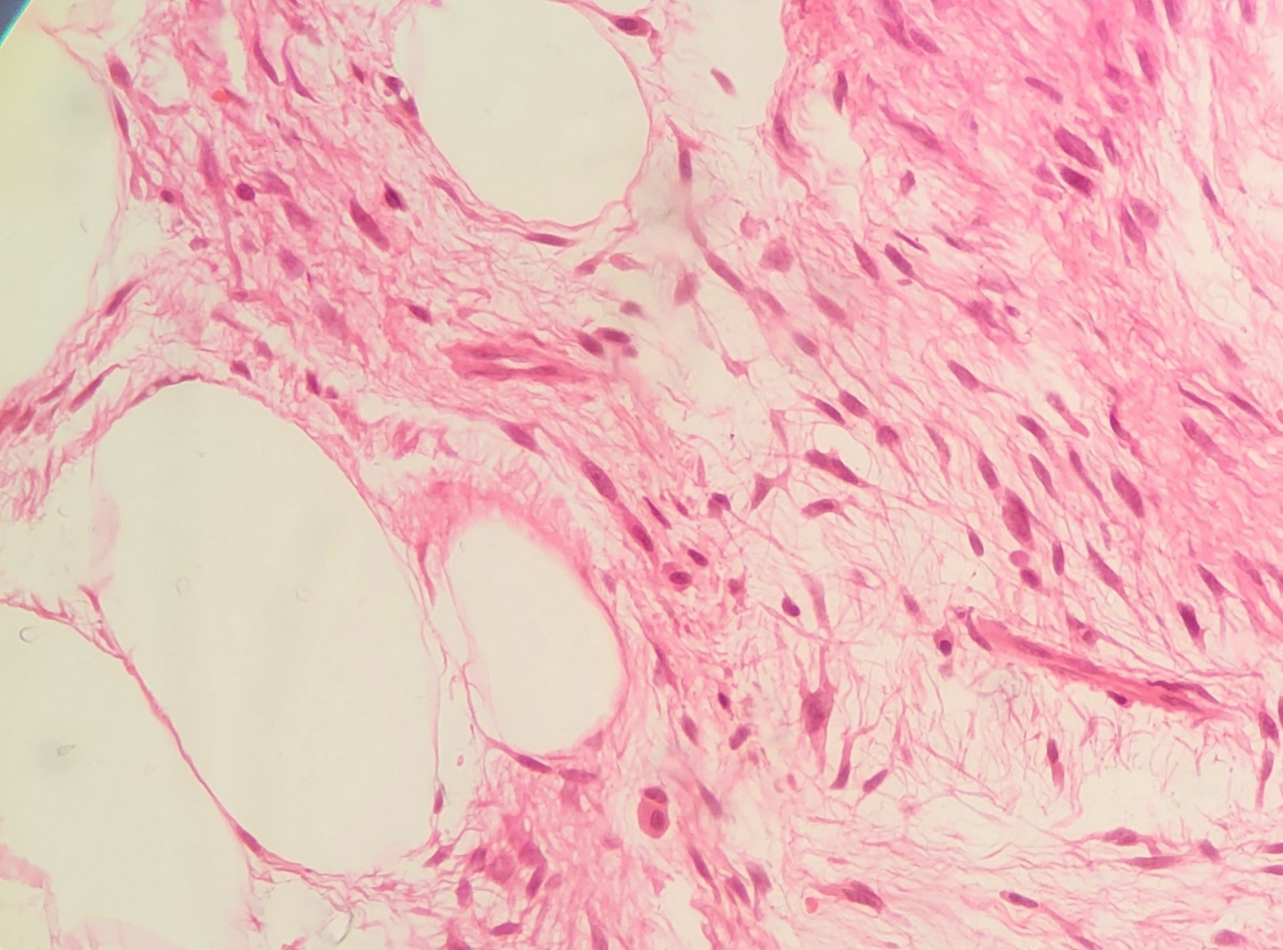
Spindle cell lipoma

**Figure 2:**
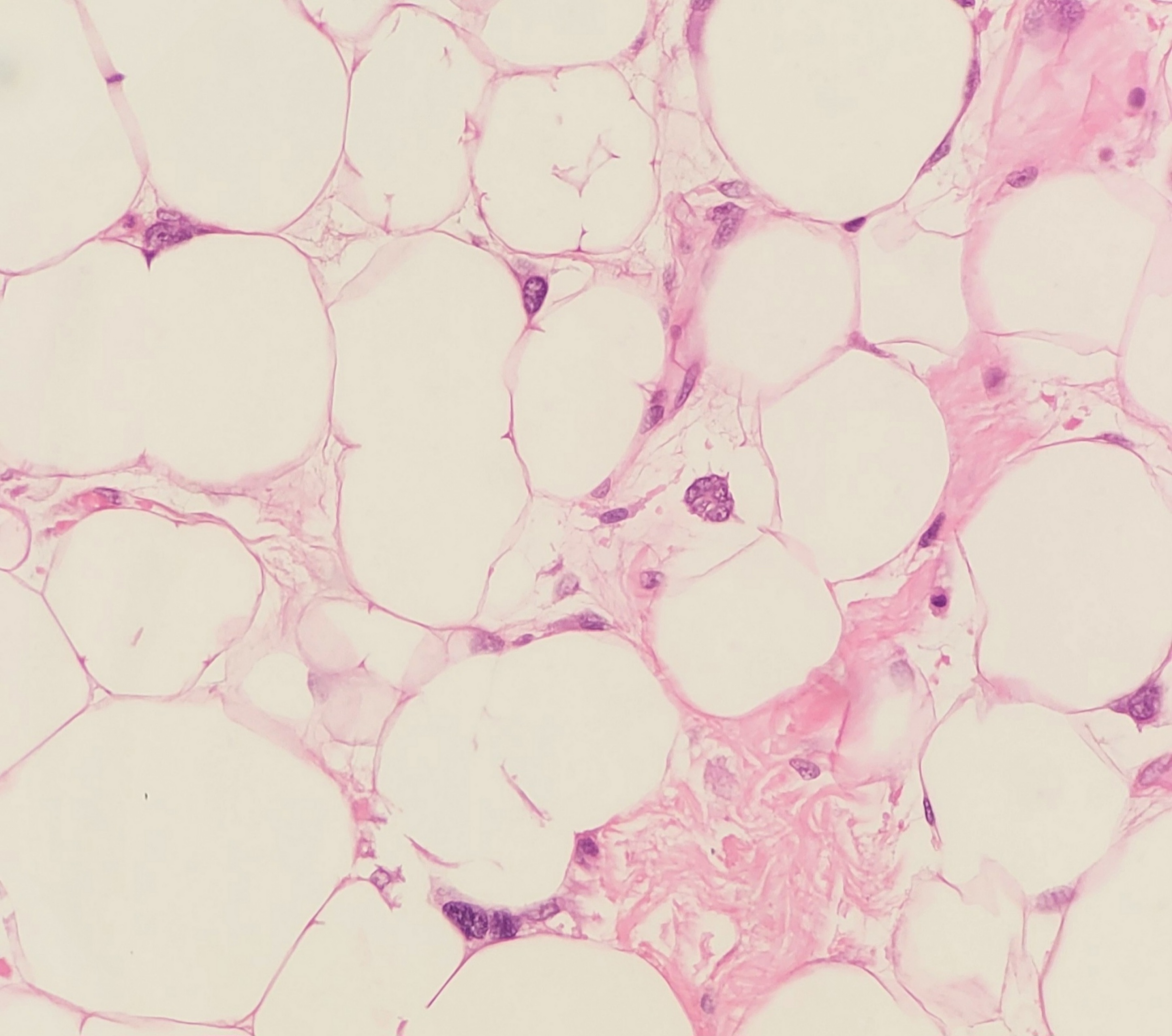
WDL with lipoblasts present WDL, well-differentiated liposarcoma

Figure 1 and Figure 2 show spindle cell lipoma and WDL with lipoblasts present.

Molecular analysis supported these findings, with MDM2 and CDK4 positivity observed in ALT/WDL and dedifferentiated liposarcoma, reinforcing the role of combined radiological, histopathological, and molecular assessment in accurate diagnosis and risk stratification.

The immunohistochemical findings are summarized in Table 6. MDM2/CDK4 immunohistochemistry was performed in 8 selected cases with diagnostic difficulty. Among these, four cases morphologically diagnosed as WDL demonstrated positivity for MDM2/CDK4, whereas four cases diagnosed as lipomatous neoplasm showed negative staining. The findings support the diagnostic utility of MDM2 and CDK4 in differentiating ALT/WDL from benign lipomatous tumors.

**Table 6:** MDM2/CDK4 immunohistochemical findings in lipomatous tumor WDL, well-differentiated liposarcoma

Histological category	Number of cases	MDM2/CDK4 expression	Diagnostic significance
WDL	4	Positive in all cases	Indicates atypical/malignant adipocytic neoplasm
Lipomatous neoplasm	4	Negative in all cases	Consistent with a benign lesion

## Discussion

Lipomatous tumors constitute a heterogeneous group of mesenchymal neoplasms ranging from benign lipomas to malignant liposarcomas. In the present study, benign lipomas accounted for the majority of cases (63%), with a smaller proportion of variants and malignant counterparts such as ALT and WDL. Similar observations were reported by Mentzel and Fletcher, who described lipoma as the most common adipocytic tumor encountered in routine pathological practice [11]. In our study, conventional lipoma represented the predominant histological subtype, reinforcing the established literature regarding the predominance of benign adipocytic neoplasms [1,11].

The age distribution in our study showed a peak incidence in the 41-60 years age group, which is comparable to the findings of Gaskin and Helms, who demonstrated that lipomatous tumors are more common in middle-aged and older adults [9]. A male predominance of 60% was observed in the present study. Kransdorf also reported a slight male predominance in soft tissue tumors, although some previous studies have shown no significant gender difference [10]. The variation between studies may be attributed to regional differences, referral patterns, and sample size.

With respect to anatomical distribution, the upper extremity was the most common site in our study, followed by the lower extremity and trunk. Kransdorf et al. and Murphey et al. similarly observed that lipomatous tumors most frequently involve the extremities and superficial soft tissue [8,12]. In the present study, the majority of benign lipomas were located in subcutaneous tissue, whereas malignant tumors were more commonly identified in deep soft tissue and intramuscular locations. Dalal et al. demonstrated that deep-seated lesions, particularly retroperitoneal and intramuscular tumors, are associated with a higher risk of malignancy and recurrence [6].

The present study showed that subcutaneous tumors constituted the majority of benign lesions, while intramuscular and deep-seated tumors were relatively fewer but demonstrated a greater association with atypical and malignant behavior. Similar findings were reported by Crago and Singer, who observed that well-differentiated and dedifferentiated liposarcomas are more frequently encountered in deep anatomical locations [7]. In our study, deep soft tissue and retroperitoneal lesions constituted 9% of cases, and a substantial proportion of these lesions showed atypical or malignant histology.

The histopathological spectrum observed in this study reflects the diversity of adipocytic neoplasms. Variants such as fibrolipoma and angiolipoma formed a significant proportion of benign tumors, consistent with the observations of Mentzel and Fletcher [11]. ALT and WDL together constituted approximately 9% of the total cases in our study. Kilpatrick et al. highlighted the diagnostic difficulty in differentiating ALT/WDL from benign lipomas based on morphology alone, particularly in borderline lesions [13]. Therefore, ancillary techniques assume significant importance in establishing the diagnosis.

Tumor size emerged as a major predictor of malignancy in the present study. Most benign tumors measured less than 5 cm, whereas malignant tumors were more commonly associated with tumor sizes greater than 10 cm. In the study by Kransdorf, larger tumor size and deep location were strongly associated with malignancy in soft tissue neoplasms [10]. Similarly, Dalal et al. demonstrated that increasing tumor size corresponds with poorer prognosis and greater likelihood of atypical or malignant pathology [6]. In our study, among tumors larger than 10 cm, malignant tumors constituted nearly half of the cases, further supporting the significance of tumor size as a predictive factor.

Radiological evaluation demonstrated an important role in differentiating benign from malignant lipomatous tumors. Kransdorf et al. reported that benign lipomas typically demonstrate homogeneous fat density and thin septations on imaging, whereas atypical and malignant tumors exhibit thick septa, nodularity, non-adipose areas, and enhancement [8]. Similar findings were observed in our study, where malignant tumors showed radiological heterogeneity, thickened septa, and deep location. Murphey et al. also described the role of MRI in identifying aggressive features in liposarcomas and aiding preoperative diagnosis [12].

Molecular and immunohistochemical analysis using MDM2 and CDK4 has become increasingly important in the diagnosis of ALTs and WDLs. Thway et al. demonstrated that MDM2 and CDK4 positivity are highly useful in distinguishing ALT/WDL from benign lipomas [3]. Similarly, Binh et al. reported strong nuclear positivity for MDM2 and CDK4 in atypical and malignant lipomatous tumors, while benign lipomas remained negative [5].

In the present study, MDM2/CDK4 immunohistochemistry was performed in selected diagnostically challenging cases. A total of eight cases underwent MDM2/CDK4 evaluation. Among these, four cases diagnosed morphologically as WDL showed MDM2/CDK4 positivity. In contrast, cases morphologically favoring benign lipoma demonstrated negative staining for MDM2 and CDK4. These findings correlate well with previous studies by Thway et al., Weaver et al., and Binh et al., who demonstrated that MDM2 amplification and CDK4 overexpression are characteristic features of ALT/WDL and are generally absent in benign lipomas [3-5].

Weaver et al. demonstrated that FISH for MDM2 amplification serves as a highly sensitive and specific method for distinguishing ALT/WDL from benign lipomatous tumors [4]. Although FISH analysis was not performed uniformly in the present study because of resource limitations, immunohistochemical positivity for MDM2/CDK4 in morphologically suspicious cases significantly aided accurate diagnosis. In our study, all morphologically confirmed WDL cases that underwent immunohistochemistry demonstrated MDM2/CDK4 positivity, while benign lipomas remained negative, supporting the diagnostic utility of these markers.

The findings of the present study reinforce the importance of integrating clinical, radiological, histopathological, and molecular parameters in the evaluation of lipomatous tumors. Doyle, in the updated WHO classification, emphasized the importance of combining morphology with molecular alterations for accurate tumor classification [14]. Our findings similarly demonstrate that tumor size, anatomical location, radiological heterogeneity, and MDM2/CDK4 expression collectively contribute to the differentiation between benign and malignant adipocytic neoplasms.

Overall, the present study corresponds well with previously published literature regarding the clinicopathological spectrum of lipomatous tumors. The predominance of benign lipomas, the increased observations of larger and deep-seated tumors with malignancy, and the diagnostic significance of MDM2/CDK4 positivity in ALT/WDL are findings that parallel earlier studies [3,6,8]. The study further highlights the importance of ancillary diagnostic techniques in resolving difficult cases and ensuring accurate classification and management.

This study has certain limitations. Being a retrospective, single-center study, the findings may not be fully generalizable to the broader population. The reliance on archived data may have led to incomplete clinical or radiological information in some cases. Advanced molecular diagnostic techniques such as MDM2 amplification studies were not performed uniformly across all cases, which could have further strengthened diagnostic accuracy, particularly in borderline lesions. Additionally, long-term follow-up data were not available, limiting the ability to assess recurrence rates and clinical outcomes.

## Conclusions

This study highlights the clinicopathological spectrum of lipomatous tumors in a tertiary care setting, reaffirming that benign lipomas constitute the majority of cases. Among the variants, fibrolipoma and angiolipoma were the most frequently encountered, while ALT and WDL represented a smaller but clinically significant subset. The findings demonstrate that increasing tumor size is associated with a higher likelihood of malignancy, with larger, deep-seated lesions showing a greater propensity for atypical or malignant behavior. Accurate diagnosis of lipomatous tumors requires a comprehensive approach integrating clinical, radiological, and histopathological evaluation. Careful assessment of larger tumors and the use of adjunct diagnostic techniques are essential, particularly in differentiating benign lipomas from ALT/WDLS, which have important therapeutic and prognostic implications.
